# Observations on the Use of Large Doses of Acetate of Lead in Fever

**Published:** 1840

**Authors:** A. Kimball

**Affiliations:** Macon County, Alabama


ART. XI.—*Observations on the use of large doses of the Acetate of Lead in some of the diseases of Alabama and Georgia.* In a letter to Dr. Drake, from A. KIMBAL, M. D., of Macon County, Alabama.

ELEVEN years have just elapsed, since I left your lecture room, and notwithstanding the ability with which you discussed the pathology, and treatment of diseases; it has been my misfortune in many cases to witness the inefficiency of many of the modes of practice pursued in diseases of this region of country—Georgia and Alabama.

Our climate is more unsteady, and the changes of temperature more frequent than in yours, (Ohio.) I have also remarked that rainy summers, are decidedly the most sickly, and the fevers more violent and unyielding. The ordinary intermittents, are not generally very difficult to arrest. A few active mercurial cathartics, or as many emetics followed by large doses of sulphate of quinine, commencing a few hours before the expected paroxysm, and at given intervals of thirty to sixty minutes, are, in most cases, sufficient. But when they assume a remitting, or continued form, with a white or yellowish fur on the tongue, loss of appetite, great thirst, headach, and general heat of body, with restlessness,

&c., the treatment must be different. Such cases generally continue from nine to fifteen days. The most difficult and unyielding form, is that which has a typhoid termination. From the seventh to the tenth day, they seem to promise a fair and happy issue; when we witness a spontaneous removal of the scurf from the tongue, the surface of which becomes red, and then dries and cracks, and the thirst increasing, and red or livid spots appearing on the tongue, with obtuseness of sensation, coma and mild delirium. Such cases had so frequently gone on to a fatal termination, in defiance of every plan of treatment laid down in the books, that I was induced in 1830, to try active bloodletting, thereby reducing as much as practicable the febrile excitement, and gaining as much remission as possible, for the free administration of calomel and quinine. The success of that plan, although more satisfactory, was not as great as was desirable; and while deeply reflecting on the inadequacy of the usual treatment in our southern fevers, I was interested with some of the items in the new French doctrine, which I believed pathologically correct, yet practically wrong. This together with Currie's reports, and his professed success in the external application of cold water to the cure of fever, induced me to suppose with others, that the mucous tissue or nervous coat of the *primæ viæ* was the seat or radiant point of fever. In connexion with this I reflected on the influence of certain cooling and sedative remedies in external injuries, such as fractures, sprains, &c., where great fever, and irritability were developed. In such cases no local application has been of greater efficacy than the solution of acetate of lead. When externally applied, if its effects be to ease pain, by reducing inflammation and quieting the irritability of the parts, then if internally used, so that its application, to the inflamed tissue should be general, would it not be a happy and salutary adjuvant in the treatment of fevers? Acting upon this suggestion, I determined to give it a fair trial. Governed by the generally received opinion of the dangerous consequences of long retention of the article in the stomach and bowels; I was induced to unite with it such purgatives as would insure its speedy transition through the alimentary canal.

In August, 1835, I was with such a case as I have described. The patient was a female, on whom I had exhausted every professional means in my power; her tongue was parched, encrusted and cracked; her thirst insatiable, &c., &c. I prepared eight powders, each containing eight grains of acetate of lead, three grains of calomel, and one-fourth of a grain of tartar emetic, and directed that the patient should take one every two hours; but should any number purge four or five times, to discontinue them. I promised to see her the ensuing day, but had no expectation of finding her alive. I was, however, agreeably surprised, to find her cool and quiet, her thirst allayed, and her tongue moist. She had taken all the powders, containing sixty-four grains of the acetate, in sixteen hours; they had operated eight times, and her thirst moderated from the exhibition of the first powder. The termination of this case was so highly satisfactory, that I prosecuted it in other cases, and that too with a success which I had not before witnessed. I suggested it to Dr. Crawford of Georgia, whose experience and professional reputation entitled his opinion to the most respectful consideration. But his over caution about lead cholic, detered him from a trial. Professor Chapman places it with the precarious articles in intermittents, but informs us that it has been successfully used in *hectic and yellow fever*, by Dr. Irving of Charlestown. He however does not tell us his mode of prescribing it, yet says, that two grains with one fourth of a grain of opium, in pills, is applicable to most cases. I have not found it so, I have not used above ten grains every two hours. But my own opinion is, and should I take the opinion of the distinguished professor for authority, I should conclude, that large doses possess a purgative effect—I have derived more benefit from eight grains, than I have ever been able to procure from six, and farther, I never use the opium in fevers, (for very obvious reasions) unless in very irritable states of the stomach and then I prefer to increase the calomel, exclude the tartar emetic, and control tne gastric excitability by sinapisms or blisters. I always add or exclude the antimony, according to the state of the bowels; I most generally trust to active purging, bleeding &c., in the inflammatory stage of the fever. If they do not succeed, I prescribe calomel, sixteen to twenty-four grains, acetate of lead, forty grains, and tartar emetic, two grains, mixed and made into powder. Of these, I direct one every two hours if the bowels are easily moved, if not, every hour or hour and a half—if they do not produce too much nausea. If they are not sufficiently operient, I follow them with oil or emenreta, or both. It is most desirable that purging should succeed the fourth or fifth, and the calomel and antimony when properly proportioned and timed, will effect that object. A fever of the same type prevails among children, in the limestone (rosten) districts of Alabama, with great fatality, often manifesting all the symptoms, usually attending the existence of worms, which symptoms arise, as Dr. Dewes, very correctly supposes, from the corroding influence of *acrid bile* on the mucus tissue of the bowels; for I confess I never have been able to expel worms under such circumstances, in sufficient numbers, to infer reasonably, their influence in producing the fever. To children I generally give two to three grains of the acetite, two grains calomel, one-eighth tartar emetic, every two hours.

Diarrhœa and dysenterics are frequent and dangerous among children, and I have not derived greater advantages from any course than the forgoing, by excluding the antimony and substituting morphium, Dover's powder, or opium every two or three hours. The same treatment I have found appliable to adults, I have tried the same medicine in a few cases of cholera morbus, with equal success, the most inveterate case which I recollected to have seen, occurred in a chief of the Creek Indians, after those around him had expendid their efforts, as well medicinal as superstitions—I prevailed on the wife who spoke English, to try the "white mans" medicine. The patient was in the last extremity, but the case very soon assumed a favorable aspect, and recovered, followed, under the use of the acetite and Dover's powder. I have had father trials, in which its efficacy is fully established. I think it best, always to have the bowels re-opened in sixteen or twenty hours after the sessation of the discharges.

In bilious plurisies, and pnumonia typhodes, I have had ample experience, sufficient to establish its efficacy. I do not entertain the belief that the acetate possesses any expectorant property, or any direct action on the liver, but it has the power, in combination with other articles, of diminishing the heat and contracting the irritability of the bowels and stomach, whereby it reduces excitement, and regulates the morbid secretions, as well petorial as abdominal.

Of its efficacy in small-pox, I can speak but imperfectly, having seen but one case, which occurred in my practice in 1836, on the Tallapoosa river, in which its beneficial influence was happily displayed.

No where have I seen its powers more decidedly manifested, than in combating the dangerous disorders, arising from the indiscriminate use of "No. 6," "lobelia," &c., &c., by the Thompsonians; who in excess of excitement prescribe active stimulants to force the *vis medicatrix* to expel the *morbid humors.*

In these cases the abdomen is often tumid, the mucous tissue of the stomach and bowels, is in a *scorched*, corroded and shattered state, from the free use of the exciting article called "No. 6," composed of Cayenne pepper, ginger, &c., infused in rectified spirits. In such cases I have found the acetate display its greatest powers.

*Modus Operandi.*—I cannot suppose that the beneficial effects of the acetate arise from its purgative property. It acts in three efficacious modes. 1st. As an astringent, by placing a proper restrictive influence over the capillaries, and controlling the inordinate alvin secretion. 2d. As a tonic, giving tone and healthy action to the emunctories. 3d. As a refrigerant; while the purgative articles combined with it expel the morbid secretions, and disgorge the glandular apparatus. I can only, sir, add, that three years experience does not enable me to say, that I have ever seen the first symptom of colic, or the least unpleasant consequence follow its application; but on the other hand, I have had every reason to place the most implicit confidence in its powers. I cannot say what effect larger doses of the article would have in the treatment of fever; I will leave that for other and abler hands. This much however I can say, that I have prescribed from five to eight grains as described in this plan, until sufficient purgation is procured, or the whole taken—this plan I have continued for four days in succession, with the desired effect.

